# Selenium Fortification Alters the Growth, Antioxidant Characteristics and Secondary Metabolite Profiles of Cauliflower (*Brassica oleracea* var. *botrytis*) Cultivars in Hydroponic Culture

**DOI:** 10.3390/plants10081537

**Published:** 2021-07-27

**Authors:** Mahboobeh Saeedi, Forouzandeh Soltani, Mesbah Babalar, Fatemeh Izadpanah, Melanie Wiesner-Reinhold, Susanne Baldermann

**Affiliations:** 1 Department of Horticultural Science, University of Tehran, Daneshkade Str., Karaj 31587-77871, Iran; mahboobeh_saedi@ut.ac.ir (M.S.); mbabalar@ut.ac.ir (M.B.); 2 Food Chemistry, Institute of Nutritional Sciences, University of Potsdam, Arthur-Scheunert-Allee 114-116, 14558 Nuthetal, Germany; izadpanah@igzev.de (F.I.); Susanne.Baldermann@uni-bayreuth.de (S.B.); 3 Leibniz-Institute of Vegetable and Ornamental Crops, Theodor-Echtermeyer-Weg 1, 14979 Großbeeren, Germany; Wiesner@igzev.de; 4 Food Metabolome, Faculty of Life Sciences: Food, Nutrition, Campus Kulmbach, University of Bayreuth, Fritz-Hornschuch-Straße 13, 95326 Kulmbach, Germany

**Keywords:** antioxidant, floret, glucosinolate, photosynthetic pigments, selenium

## Abstract

Nowadays the importance of selenium for human health is widely known, but most of the plants are poor in terms of selenium storage and accumulation because of the low selenium mineralization potential of the soil. For this purpose, foliar application of different sodium selenate concentrations (0, 5, 10, 15, 20 mg/L) was used to treat the cauliflower cultivars “Clapton” and “Graffiti”. Higher yields and other related vegetative attributes were improved at 10 and 15 mg/L sodium selenate application. At a concentration of 10 mg/L sodium selenate, photosynthetic pigments, total phenolic compounds and antioxidant capacity were enhanced in both cultivars, but the “Graffiti” cultivar responded stronger than the “Clapton” cultivar. The glucosinolates were accumulated in response to selenium fortification and the highest amounts were found in the “Graffiti” cultivar at 10 mg/L. Selenium accumulated concentration-dependently and rose with higher fertilization levels. In general, foliar application of selenium at 10 mg/L led to an accumulation of secondary metabolites and also positively affected the growth and yield of florets.

## 1. Introduction

Selenium (Se) is an essential element in human nutrition but, for instance, in Germany or New Zealand, Se is only present in small amounts in soils, which means that the selenium content of vegetables is correspondingly low. As a result of targeted applications of Se, the plant increasingly accumulates the mineral. The higher solubility of selenate compared to selenite plays an important role in the transport and metabolism of Se in the plant, so when the plant is treated with selenate, Se is mainly transferred to the leaves, but in a plant treated with selenite, Se is mainly accumulated in the roots and a small amount is transmitted to the aerial parts [1]. Se treatments caused a significant improvement in vegetative growth and photosynthetic pigment accumulation in cucumber and peanut plants [2,3]. Furthermore, some investigations have indicated that Se at appropriate levels can partially reduce chloroplast degradation and increase chlorophyll content [4] and has protective roles under salt and drought stress conditions [5]. In addition, Se fortification impacts positively the quality of plant based food and Se treatment enhances the content of secondary plant substances, such as phenolic compounds [6]. Se supplementation by brassica products is considered a valid method that enhances anticancer chemical compounds such as glucosinolates, phenolic compounds and carotenoids [7] in the human diet [8]. Cauliflower has various health benefits such as overcoming digestive disorders and protecting against the negative effects of ultraviolet rays, diabetes and obesity. This healthy food product is also called “Brassica box” [9]. Glucosinolates are important components in the family of Brassicaceae, which act as a natural defense in plants against herbivores. Glucosinolates are broken down during chewing and the digestion process into bioactive compounds such as isothiocyanates, which trigger detoxification processes in the human body [10].

Se is involved in the production of hormones and enzymes as well as the regulation of the immune system. Moreover, it has antioxidant, anti-inflammatory, and antiviral effects [11,12]. Se is also an integral part of some antioxidant enzymes, which protect cells from being damaged by reactive oxygen species [13]. Based on the multitude of functions, the recommended intake of Se in the diet for humans is 55 µg per day for adults according to the World Health Organization. However, the Nutrition Association of Germany, Austria and Switzerland recommend 35 to 70 µg of selenium per day for adult men and women. A tolerable dose of Se for adults is reported to be 400 µg per day [14].

Usually, the main ingestion in our diets is *via* the consumption of meat or fish. The fortification of vegetables allows vegetarians or vegans to fulfill their needs based on a plant based diet without the need for supplements. The aims of the present study were (i) to increase the Se concentration in two differently pigmented cauliflower cultivars (the “Graffiti” purple and the “Clapton” white) by foliar application and (ii) to identify the dosage of Se application for optimal growth and enrichment of value-added phytochemicals, such as glucosinolates, phenolic compounds, carotenoids and chlorophylls.

## 2. Results

### 2.1. Growth Parameters

Flower formation and twisting of the inner leaves, was observed about 14 days earlier in the “Graffiti” than in the “Clapton” cultivar at 10 and 15 mg/L sodium selenate compared to the untreated plants. The results revealed that the “Clapton” cultivar produced heavier florets than the “Graffiti” cultivar at all sodium selenate concentrations. Treatment with 10 mg/L of sodium selenate resulted in the highest floret weight compared to the other concentrations of selenium for both cultivars. The floret of the “Clapton” cultivar was more compact than that of the “Graffiti” cultivar, and respecting floret diameter, the “Graffiti” cultivar showed wider floret than the “Clapton” cultivar. All foliar concentrations of sodium selenate improved floret diameter compared to control plants (Table 1). Measuring the root fresh weight for the “Graffiti” cultivar revealed that there were no significant differences among all sodium selenate concentrations, but it had improved compared to the control plants. Among the different concentrations of sodium selenate, the 10 mg/L increased root dry weight compared to the control plants in both cultivars. The stem diameter for both cultivars increased slightly at 10 mg/L of sodium selenate application, though there was no difference for all treatments for the stem height in the “Clapton” cultivar, but we saw an increase for the “Graffiti” cultivar at all Se concentrations (Table 1).

Leaf traits were affected by cultivar types and sodium selenate concentrations. In this study, the “Graffiti” cultivar showed an increase in leaf fresh weight from 575.75 g in control plants to 728.93 g when treated with 10 mg/L of sodium selenate (increase 26%). Similar results were observed for the dry weight of the leaves at 10 mg/L of sodium selenate which induced a 33% increase (41.4 g compared to 55.21 g control plants) (Table 2). Moreover, a significant increase in leaf area from 455 cm^2^ in control plants to 535 cm^2^ (17% increase) was observed at 15 mg/L of sodium selenate application for the “Graffiti” cultivar. The root growth was increased at 10 mg/L sodium selenate for both cultivars compared to control treatment, which is also reflected the increase in root lengths. Means of roots length for the “Graffiti” and the “Clapton” cultivars without application of sodium selenate were 36.88 and 34.58 cm, whereas this reached to 42.22 cm and 40.66 at 10 mg/L, respectively (Table 2).

### 2.2. Changes in Photosynthetic Pigments, Antioxidant Capacity, Phenolic Compounds and Selenium Content in the Floret and the Leaves

The amount of photosynthetic pigments, chlorophyll a, b and carotenoid contents of leaves significantly increased with sodium selenate application in both cultivars compared to the control plants (Table 3). In general, the “Clapton” cultivar accumulated more carotenoids and chlorophyll than the “Graffiti” cultivar (Table 3). However, among Se treatments, higher levels of sodium selenate (20 mg/L) led to lower pigment concentrations compared to 10 and 15 mg/L foliar treatment in both cultivars. The antioxidant capacity increased approximately 40 % at the concentration of 5 mg/L of sodium selenate for the “Graffiti” cultivar compared to the control. Sodium selenate application resulted in the accumulation of polyphenolic compounds in the florets of the “Graffiti” rather than of the “Clapton” cultivar and highest accumulation was observed after treatment with 10 and 15 mg/L of sodium selenate for both cultivars. The two cauliflower cultivars accumulated Se in a concentration-dependent manner. The highest Se accumulation was found after treatment with 20 mg/L sodium selenate and was 0.57 mg/kg in the “Clapton” and 0.44 mg/kg in the “Graffiti” florets (Table 3), whereas improved growth performance and enhanced accumulation of phytochemicals was observed at 10 and 15 mg/L.

### 2.3. Glucosinolate Compounds in Leaves

**Figure 1:**
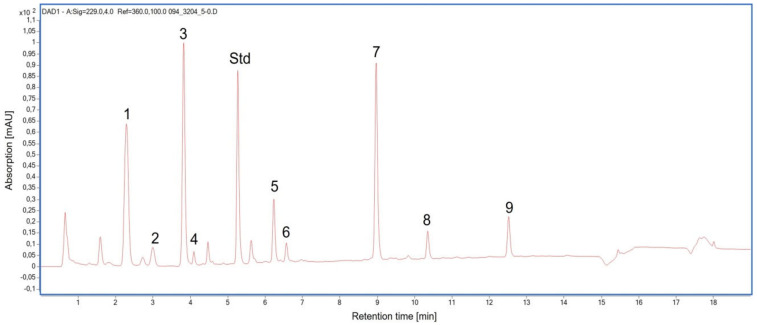
Chromatogram at 229 nm of the glucosinolate analysis of a cauliflower floret. Std, internal standard; 1, 3-methylsulfinylpropyl glucosinolate; 2, (*R*)2-hydroxy-3-butenyl glucosinolate; 3, 2-propenyl glucosinolate; 4, 4-methylsulfinylbutyl glucosinolate; 5, 4-hydroxyindol-3-ylmethyl glucosinolate; 6, 3-methylthiopropyl glucosinolate; 7, indol-3-ylmethyl glucosinolate; 8, 4-methoxyindol-3-ylmethyl glucosinolate; 9, 1-methoxyindol-3-ylmethyl glucosinolate.

In both cauliflower cultivars, in total the following glucosinolates (GS) were determined (Figure 1): the aliphatic GS 3-methylthiopropyl GS, 3-methylsulfinylpropyl GS, 4-methylsulfinylbutyl GS, 2-propenyl GS, and (*R*)2-hydroxy-3-butenyl GS, as well as the indolic GS indol-3-ylmethyl GS, 4-hydroxyindol-3-ylmethyl GS, 4-methoxyindol-3-ylmethyl GS and 1-methoxyindol-3-ylmethyl GS.

**Figure 2:**
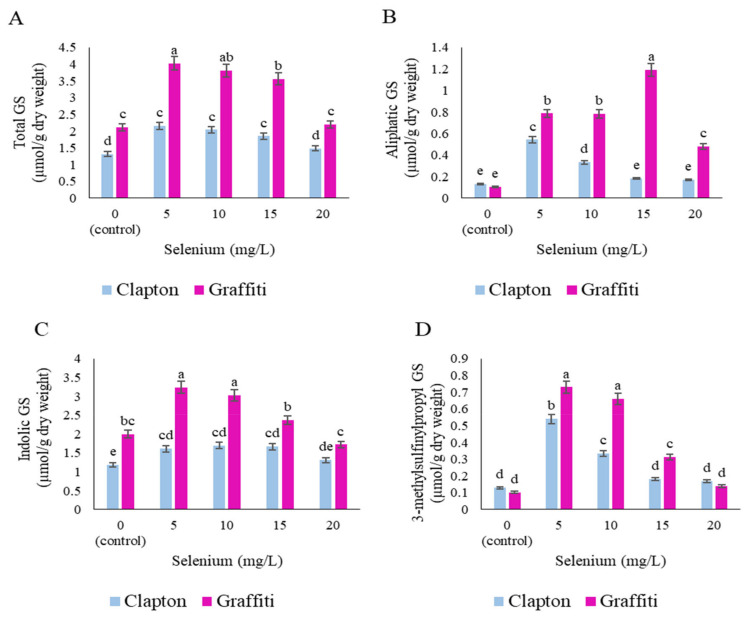

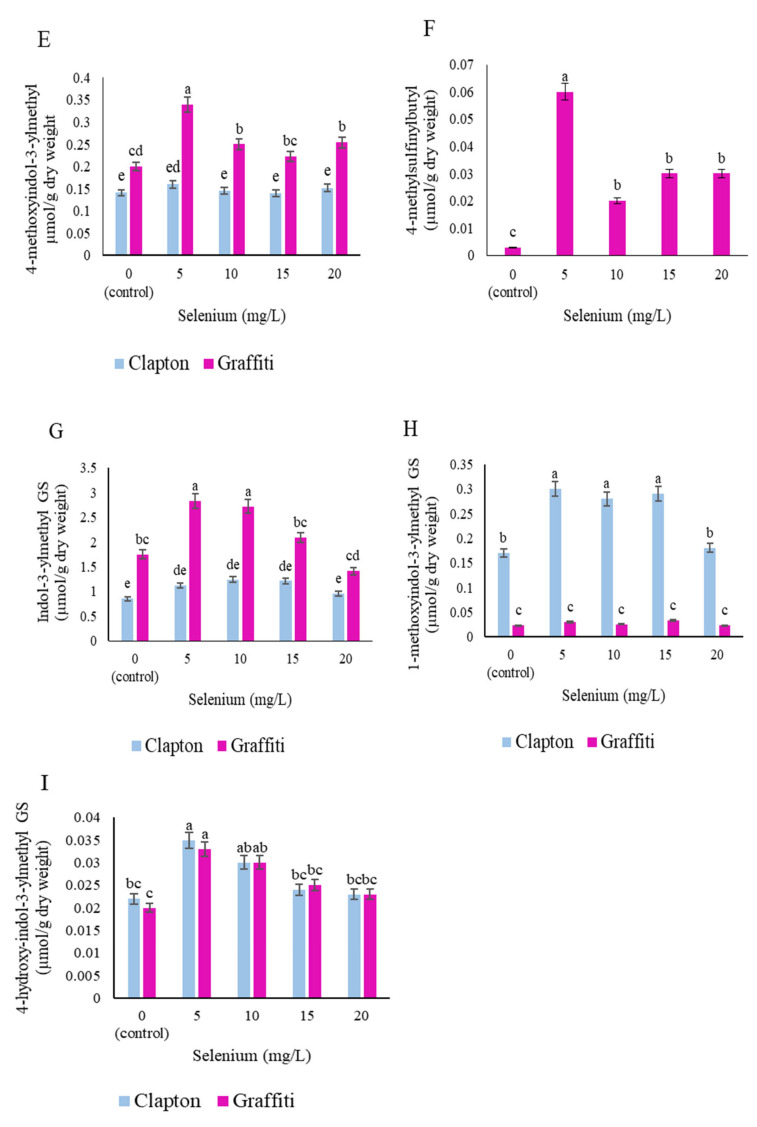
Mean comparison of sodium selenate treatments on glucosinolate (GS) of cauliflower leaves (“Clapton, “Graffiti”). (**A**) Total GS, (**B**) Aliphatic glucosinolates, (**C**) Indolic glucosinolates, (**D**) 3-methylsulfinyl-propyl GS, (**E**) 4-methoxyindol-3-ylmethyl GS, (**F**) 4-methylsulfinylbutyl GS, (**G**) indol-3-ylmethyl GS, (**H**) 1-methoxyindol-3-ylmethyl GS and (**I**) 4-hydroxy-indol-3-ylmethyl GS. Shown are the mean ± standard deviations of three replicates. Bars marked by the same letter are not significantly different (*p* ≤ 0.05, Duncan’s multiple range tests).

The total GS content was in general of a higher amount in the “Graffiti” cultivar than in the “Clapton” cultivar. At a treatment concentration of 5 mg/L of sodium selenate, the “Graffiti” cultivar accumulated nearly twofold more than the “Clapton” cultivar at the same concentration. Application of sodium selenate at 5 and 10 mg/L affected total GS more than the other Se treatments (Figure 2A) in both cultivars. In contrast, higher concentrations of sodium selenate (20 mg/L) led to a significant decrease of the total GS (Figure 2A). Higher concentrations of aliphatic GS were detected at 5 mg/L of sodium selenate in the “Clapton” cultivar and for the “Graffiti” cultivar at 15 mg/L. Indolic GS of the “Graffiti” cultivar increased significantly at 5 and 10 mg/L sodium selenate (62 and 51%, respectively) and decreased at 20 mg/L (14%) compared to control plants (Figure 2B,C).

Leaf samples of the “Graffiti” cultivar revealed higher contents of 3-methylsulfinylpropyl GS (Glucoiberin) and 4-methoxyindol-3-ylmethyl GS (4-methoxy glucobrassicin) in samples treated with 5 mg/L sodium selenate (Figure 2). The amount of 4-methoxyindol-3-ylmethyl GS in the “Clapton” cultivar was not statistically significant between sodium selenate treatments and control plants (Figure 2D,E). 4-methylsulfinylbutyl GS (Glucoraphanin) was not detected in the “Clapton” cultivar leaves and, for the “Graffiti” cultivar, its content increased sharply by 0.057 µmol/g DW more than control plants at 5 mg/L of sodium selenate treatments (from 0.003 in control plants to 0.06 at 5mg/L) (Figure 2F). Indol-3-ylmethyl GS (Glucobrassicin) was found to be significantly increased by sodium selenate treatment with 5 and 10 mg/L (Figure 2G). The responses of cultivars were different and “Graffiti” cultivar accumulated more than the “Clapton” cultivar. Changes in the indol-3-ylmethyl GS content in the “Clapton” leaves were not significant, but an increasing trend was found at all foliar treatment concentrations (Figure 2G). In contrast, 1-methoxyindol-3-ylmethyl GS (Neoglucobrassicin) increased in “Clapton”, and in the “Graffiti” cultivar there was no significant variation even in treated plants by sodium selenate and controls (Figure 2H). As it illustrated in Figure 2I the amount of 4-hydroxyindol-3-ylmethyl GS (4-hydroxy glucobrassicin) was significantly increased (70%) compared to control plants after treatment with 5 mg/L sodium selenate for both cultivars and 10 mg/L had a similar effect (Figure 2I). 

### 2.4. Glucosinolate Compounds in the Floret

**Figure 3:**
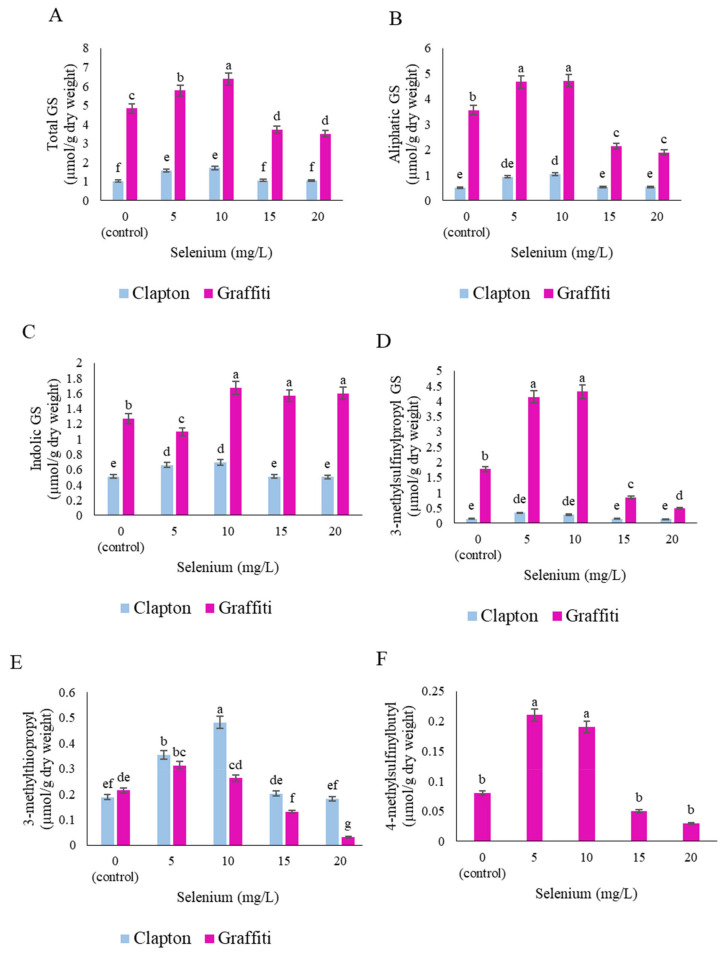

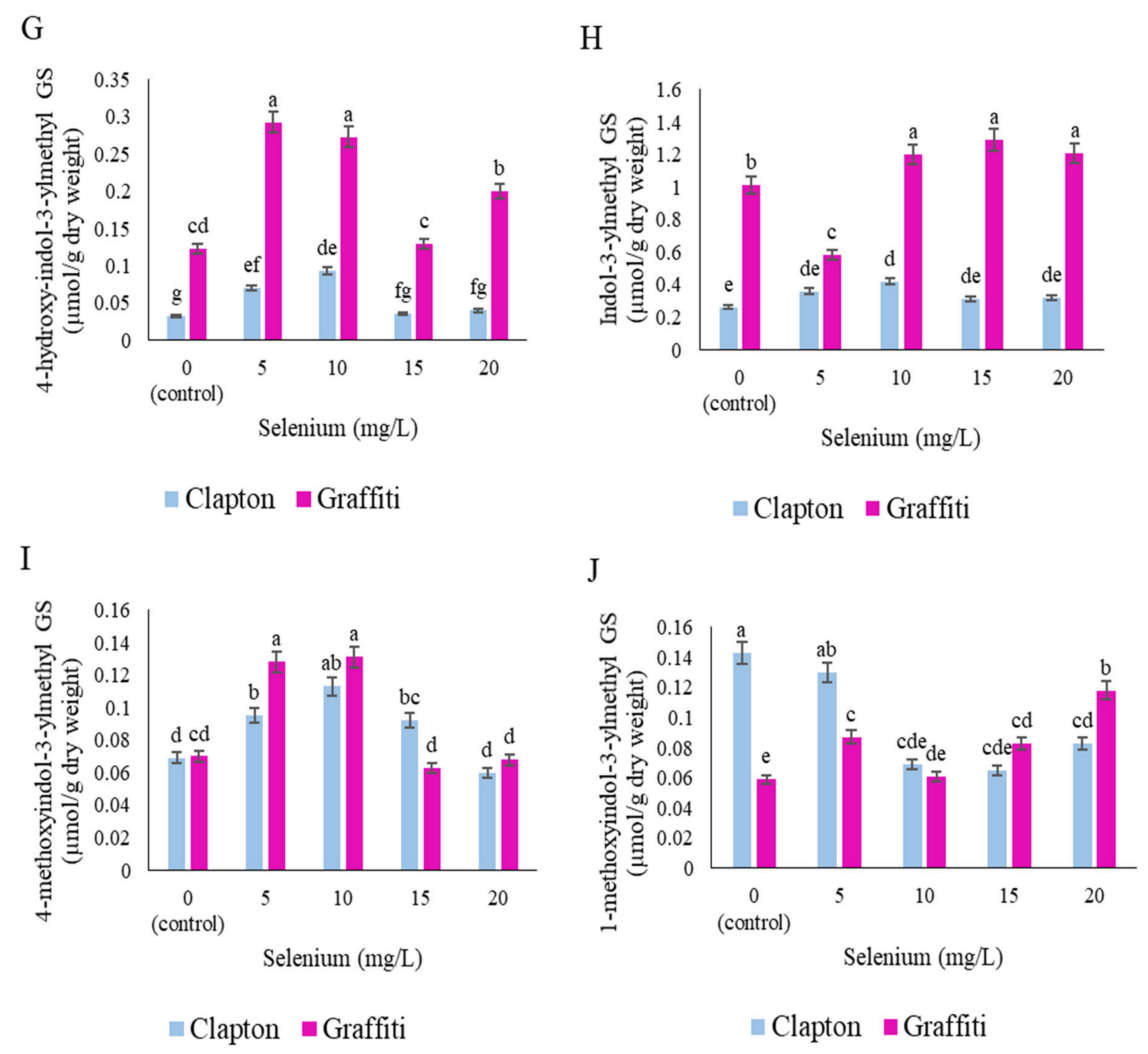
Mean comparison of sodium selenate treatments on glucosinolate (GS) profile of cauliflower florets (“Clapton, “Graffiti”), (**A**) Total GS, (**B**) Aliphatic glucosinolates, (**C**) Indolic glucosinolates, (**D**) 3-methylsulfinylpropyl GS, (**E**) 3-methylthiopropyl, (**F**) 4-methylsulfinylbutyl GS, (**G**) 4-hydroxyindol 3-ylmethyl GS, (**H**) indol-3-ylmethyl GS, (**I**) 4-methoxyindol-3-ylmethyl GS, (**J**) 1-methoxyindol-3-ylmethyl GS. Shown are the mean ± standard deviations of three replications. Bars marked by the same letter are not significantly different (*p* ≤ 0.05, Duncan’s multiple range tests).

The total GS was more in the “Graffiti’ florets than the “Clapton” cultivar and treated plants with 10 mg/L of sodium selenate increased by 32% compared to the control for both cultivars (Figure 3A). Aliphatic glucosinolates were raised after application of 5 and 10 mg/L of sodium selenate in both cultivars, more than treated plants (Figure 3B). Indolic glucosinolates were increased more in the “Graffiti” than in the “Clapton” cultivar, at concentrations of 10 to 20 mg/L compared to 5 mg/L of sodium selenate (Figure 3C).

The 3-methylsulfinylpropyl GS (Glucoiberien) detected for the “Graffiti” cultivar was highly significant at 5 and 10 mg/L vs the same concentration in the “Clapton” cultivar. However, 5 and 10 mg/L of sodium selenate enhanced the accumulation of 3-methylsulfinylpropyl GS in the “Graffiti” cultivar, but in samples treated with 15 and 20 mg/L the 3-methylsulfinylpropyl GS content was reduced to 53% and 73%, respectively (Figure 3D). Application of sodium selenate at concentration of 10 mg/L on the “Clapton” cultivar gave a greater amount of 3-methylthiopropyl GS (Glucoibeverin), whereas the “Graffiti” cultivar tended to accumulate less. 3-methylthiopropyl GS in both cultivars was reduced at 20 mg/L and sharply declined for the “Graffiti” cultivar by around 86% vs control plants (Figure 3E). An interesting result was obtained for 4-methylsulfinylbutyl GS (Glucoraphanin) in the “Clapton” cultivar in which 4-methylsulfinylbutyl GS was below the detection limit at all sodium selenate concentrations, whereas in the “Graffiti” cultivar 4-methylsulfinylbutyl GS increased 0.13 µmol/g DW and 0.11 µmol/g DW more than control plants at 5 and 10 mg/L of sodium selenate foliar application, respectively (Figure 3F). Sodium selenate application affected, in both cultivars, the content of 4-hydroxyindol-3 yl-methyl GS (4-hydroxy glucobrassicin) at 5 and 10 mg/L sodium selenate, revealing any significant difference at those foliar Se concentrations (Figure 2G). The increase in sodium selenate concentration to more than 5 mg/L resulted in the increase of indol-3-ylmethyl GS (Glucobrassicin) production in the “Graffiti” cultivar and remained at a similar content after application at 10, 15, and 20 mg/L. The “Clapton” cultivar increase in relation to Se for indol-3-ylmethyl GS production was low at 10 mg/L of sodium selenate compared to control plants (Figure 3H).

As illustrated in Figure 3I, however, both cultivars reacted to producing higher amounts of 4-methoxyindol-3-ylmethyl GS (4-methoxy glucobrassicin) at 10 mg/L. In the case of 1-methoxyindol-3-ylmethyl GS (Neo-glucobrassicin) the reaction of cultivars to different concentration of Se treatments was different. The interaction effect of cultivars and sodium selenate concentrations indicated that the “Clapton” cultivar tended to accumulate more 1-methoxyindol-3-ylmethyl GS without treatment and after treatment with 5 mg/L of sodium selenate, while the “Graffiti” cultivar revealed higher contents at 20 mg/L than other treatments (Figure 3G).

## 3. Discussion

Se application improved the growth performance in cauliflower cultivars. With respect to sodium selenate concentrations on growth traits, 10 mg/L of sodium selenate application was the most effective and improved growth of both cultivars. An increase in root fresh and dry weight of around 20% and 28% respectively indicated that foliar application of 10 mg/L of sodium selenate improved underground growth of cauliflowers. Sun [15] stated that Se can increase the mitotic division of root tip cells in garlic and thereby increase root growth, but at higher levels it reduced cell division in these cells. It was demonstrated that Se at suitable concentrations promoted plant growth, optimal hormonal homeostasis, and nutrient partitioning within broccoli crops resulting in enhanced root growth fresh and dry weight [16]. Low concentrations of Se, possibly by increasing the amount of starch in chloroplasts, increase plant growth [15]. Se at higher concentration showed negative effects on growth parameters. Reducing the biomass of the plant by increasing the concentration of Se can be due to changes in membrane permeability to Na^+^, K^+^, and Ca^2+^ ions, causing a disturbance in respiration and water uptake [17].

An increased yield of both cultivars of around 23% at 10 mg/L sodium selenate could be attributed to an increase of photosynthetic pigments. In accordance *B. napus* cultivars responded differently against varied Se treatment levels. Lower Se level had positive effects on physio-biochemical, anatomical and molecular processes and improved photosynthetic efficiency in *B. napus* plants. Higher Se doses resulted in phytotoxicity by impairing of the physio-biochemical and molecular processes [18].

Both cultivars revealed an increase trend in antioxidant activity of florets, whereby the “Graffiti” cultivar had higher antioxidant capacity compared to the “Clapton” cultivar. This might be at least partially explained by the higher concentration of phenolic compounds in the “Graffiti” compared to the “Clapton” cultivar. It was reported that the purple Asia type cauliflower contained higher amounts of total phenolic compounds resulting in higher antioxidant activity [19]. Most phenolic compounds, including anthocyanins, are stronger antioxidants than other groups of antioxidants. Anthocyanin-rich vegetables such as purple or red colored vegetables have high antioxidant capacity [20]. A further study demonstrated that optimal Se improved the photosynthetic pigments by increasing antioxidant activity and delaying the aging of leaf tissues in kohlrabi plants (*Brassica oleracea* L. var. *gongylodes* L.) [21].

In the present study, it was found that the treatment of sodium selenate resulted in a significant increase in the total content of polyphenolic compounds in the leaves as compared to untreated plants. There is also evidence of an increase in the total phenolic compound content and anthocyanins in tomato leaves by foliar treatment and the addition of Se to nutrient medium, respectively [8]. The use of Se has also increased the amount of polyphenolic compounds in wheat. It is possible that this is the results of the increase in the activity of the enzyme phenylalanine ammonialyase (the key enzyme involved in the biosynthesis of phenolic compounds) [22]. Changes in phenolic composition content in response to Se were observed in broccoli [23], lettuce [24] and tomatoes [8], whereby phenolic composition accumulation is different and depends on species response to Se doses and the developmental stage in which it was used. The increase in chlorophyll content is the result of the protection of chloroplast enzymes by Se, which affects the oxidation/reduction state of the leaves and, thus, increases the biosynthesis of photosynthetic pigments [22,25].

In our study, the accumulation of Se in the floret of both cultivars increased by increasing the sodium selenate concentration and the “Clapton” cultivar accumulated 29.5% more than the “Graffiti” cultivar. Despite the main effects of the environment in Se accumulation, the significant genetic effects on Se concentration have been observed in broccoli florets (*B. oleracea* L. Italica Group) [26], sprouts of cauliflower (*B. oleracea* L. Botrytis Group), kale (*B. oleracea* L. Acephala Group) and Chinese cabbage (*B. rapa* L.) [27]. Plants of the Brassicaceae family are of particular importance, including the accumulation and synthesis of Se. When a plant absorbs Se, one of its possible pathways is the interlinkage to the sulfur metabolism and therefore to glucosinolate biosynthesis [10].

The “Graffiti” cultivar accumulated more total GS in both organs, laves and florets. The content of GS in plants varies between cultivars, plant individuals and plant tissues and are impacted by environment and plant nutrients [2,28]. The data from our experiment illustrated that Se enrichment influenced the “Graffiti” cultivar more than the “Clapton” cultivar in the case of aliphatic and indolic GS contents. Besides the significant variation between cultivars, the response of leaves and florets were also different. The indol-3-ylmethyl GS (Glucobrassicin) obtained more value in leaves rather than florets. Furthermore, 1-methoxyindol-3-ylmethyl GS (Neo-glucobrassicin) increased slightly more in leaves than in florets at 10 mg/L sodium selenate treatment (with no significant difference between the two cultivars). In contrast to the results for aliphatic GS which have been detected in higher values in the floret, the indolic GS were found more in leaves. An investigation of nine *Brassica* species pointed that content and composition of GS in *Brassica* vegetables depends on the tissue type as well as the plant’s genotype [29]. The same results reported for type and concentration of individual glucosinolates varied according to plant variety and plant organs of different cabbages (*B. oleracea*) [30].

Five broccoli genotypes with low and high levels of sodium selenate solution (0.17 and 5.2 mM) were fertilized with fortified nutrient solution after the emergence of florets. The results showed that the sensitivity of aliphatic glucosinolates, especially 4-methylsulfinylbutyl GS (Glucoraphanin), to Se fertilizer could be the result of reduced competition between cysteine and methionine biosynthesis due to competition between Se-cysteine and Se-methionine biosynthesis [31]. In this study clear variation in the quantities of 4-methylsulfinylbutyl GS (Glucoraphanin) between two cultivars has also been identified. Se application improved the 4-Methylsulfinylbutyl GS content of florets, but the compound was significantly influenced by the genetic control of the cultivars. 4-Methylsulfinylbutyl GS is the precursor of sulforaphane and cultivars with high content are especially valuable for healthy nutrition. A previous study also showed that purple cultivars exhibited higher contents of total GS and also 4-methylsulfinylbutyl GS than white cultivars [7].

## 4. Conclusions

This study provides more evidence of Se enrichment and its beneficial effects on plant growth and enrichment of valuable plant secondary metabolites for production of healthy vegetables. Furthermore, it can be concluded that the purple “Graffiti” cultivar had better floret quality parameters, reflected for instance by a high antioxidant capacity as well as the accumulation of aliphatic and indolic GS. Since both cultivars are prone to accumulate the Se in floret at 20 mg/L of sodium selenate application and this dosage reduced plant growth with a simultaneous decrease in phytonutrients, this could be reported as a critical dose for cauliflowers. Application of 10 mg/L of sodium selenate regarding to growth characters and accumulation of secondary metabolites and antioxidant capacity could be the proper concentration that can be used for cauliflower cultivation with enhanced selenium and secondary metabolite concentrations.

## 5. Materials and Methods

### 5.1. Materials

This research was conducted in the greenhouse of the Horticultural Science Department of the University of Tehran in 2019–2020. Plant material included two cauliflower cultivars, the “Graffiti” and the “Clapton” treated with five concentrations of sodium selenate (Na_2_SeO_4_) 0 as a control, 5, 10, 15, 20 mg/L that performed as factorial experiment with three replications and three plants in each replicate in a randomized complete block design. Seeds were planted in mid-January in beds containing coco peat and perlite at a ratio of 1:1 in 72-cell seedling trays. After emergence of true leaves, seedlings were supplied with Fusamco liquid nutrient (Yara vita, Norway) every two days and in the four true leaves stage; they were transferred to pots with a diameter of 20 and 21 cm which were filled with perlite. The distances between and within the rows were about 60 cm and 30 cm. Mean temperature at the beginning of the transfer was 17.5 °C and mean day length 10.2 h. Hoagland’s solution was initially applied in the form of a quarter, then half, and finally with complete concentration. During the growth period plants were exposed to different concentrations of sodium selenate (Na_2_SeO_4_) three times by foliar application: 6 to 8 leaves (two weeks after transfer), 12 to 14 leaves (five weeks after transfer), and 18 to 20 leaves (8 weeks after transfer). After floral harvesting, growth parameters such as floret weight and diameter, fresh and dry weight of stem, leaves and roots, stem height (from floret to crown location) and root and stem diameter were recoded. To measure the leaf area on average, three full expanded leaves from each plant were separated measured by the leaf area meter (Model: DELTA-T DEVICES, Cambridge, UK) and the data recorded in square centimeters.

### 5.2. Determination of Photosynthetic Pigments, Polyphenolic Compounds and Antioxidant Capacity

Chlorophylls a, b and carotenoid measurements were performed by homogenizing 0.5 g fresh leaves (samples of three plants in each replicate bulked) in 8 mL of 80% acetone. Absorbance of the extract at 663, 645 (chlorophyll a, b) and 510 nm (carotenoid) was measured with a spectrophotometer and total chlorophyll concentration was calculated using the formulas described by Arnon [32]. To measure the polyphenolic compounds three florets of each replicate were selected randomly and kept at −80 °C. Briefly, for analysis 0.5 g of floret samples were powdered and extracted in 80% methanol for 15 h at room temperature on an orbital shaker. Then, the extract was centrifuged and filtered through a Whatman No. 42 filter paper, and 1 mL of supernatant was mixed with 3 mL distilled water in a 15 mL falcon tube. After adding 1 mL of Folin reagent, the solution was incubated in a water bath at 27 °C for 5 min. Then, 1 mL of saturated sodium carbonate was added. After 1 h, absorbance of the extract was measured with an EONTM microplate spectrophotometer (BioTek^®^ Instruments Inc. Highland Park, Winooski, VT, USA) at 640 nm. Gallic acid standards at different concentrations (5, 10, 25, 50, 75, and 100 ppm) were used for the calibration. The amount of polyphenolic compounds was expressed in mg equivalent of gallic acid per 100 g of fresh weight [33]. The antioxidant capacity of the extracts was determined by free radical scavenging (2,2-diphenyl-1-picrylhydrazyl; DPPH). The aliquot obtained for total phenol analysis was also used for the measurement of radical scavenging activity. For this purpose, 0.01 mM DPPH solution was prepared. Then, in each sample, 0.5 mL of floret extract and 2 mL of DPPH solution were combined. After 30 min of rest in the dark, it was read at 515 nm using the method according to Koleva et al. [34]. Free radical-scavenging activity (%) was calculated using the following equation: % of DPPH radical-scavenging activity = (B − A) × 100/B where A is the absorbance of ((Sample + DPPH) − (Sample + Methanol)), and B is the absorbance of ((Methanol + DPPH) − (Methanol)). The IC50 value, which is the concentration required to obtain 50% antioxidant capacity, was calculated and used to compare the antioxidant activity of sample extracts.

### 5.3. Determination of the Total Selenium Content in the Leaves and Florets

To measure the amount of Se accumulation in the leaves and florets of cauliflower, 10 g of leaves and floret washed thoroughly distilled water to remove any dust and kept in an oven at 80 ° C for 48 h. After drying in the oven, the plant samples were pulverized and prepared by wet digestion using hydrogen peroxide (H_2_O_2_) and nitric acid (HNO_3_) to determine the total Se. One gram of the plant sample was added to the glass digestion tubes and was dissolved in 10 mL of 65% HNO_3_ and stored overnight. The next day the samples were heated at 60 °C for 30 min in a digestion block system. Then 3 mL of 30% H_2_O_2_ was added to the samples and digestion was continued at 120 °C for 90 min. After cooling, the samples were taken to a total volume of 50 mL with deionized water and finally filtered. For all samples, a digested control sample was prepared to observe possible contamination [35]. Then the amount of selenium was measured by inductively coupled plasma-optical emission spectrometry with MPX model (ICP-OES) with a wavelength of 196.026 nm.

### 5.4. Glucosinolate Compounds

To measure glucosinolate compounds, floret and leave samples were grinded with liquid nitrogen and transferred to 2 mL microtubes, lyophilized and stored in a −80 °C freezer. Briefly, 10 mg of lyophilized and homogenized plant material were extracted in presence of 0.02 µmol of the internal standard 4-hydroxybenzyl GS with hot 70% methanol (LC-MS grade, Th. Geyer GmbH & Co. KG, Renningen, Germany) and samples were prepared as described before [36,37]. The desulfo-GS were analyzed using a 1290 Infinity II UHPLC-DAD coupled with a 6230 ToF-LC/MS (Agilent Technologies, Waldbronn, Germany) with a Poroshell 120 EC-C18 column (Agilent Technologies, Waldbronn, Germany; 100 mm × 2.1 mm, 2.7 μm). UHPLC conditions were as follows: solvent A, MilliQ water; solvent B, 100% *v*/*v* acetonitrile. The 19 min run comprised 0.2% (*v*/*v*) B (2 min), 0.2% to 19.8% (*v*/*v*) B (10 min), a 2 min hold at 19.8% (*v*/*v*) B, 19.8% B to 50% (*v*/*v*) B (1 min), a 1 min hold at 50% (*v*/*v*) B, 50% to 0.2% (*v*/*v*) B (1 min), and finally a 2 min hold at 0.2% (*v*/*v*) B. The injection volume was 5 µL, and determination was conducted at a flow rate of 0.4 mL min^−1^ and 30 °C and a wavelength of 229 nm. The concentration of desulfo-GS was calculated by the peak area relative to the area of the internal standard.

### 5.5. Data Analysis

The data obtained from the experiments of this research were analyzed based on the statistical design used using SAS statistical software version 9.4 and the comparison of the mean was performed using Duncan’s multiple range tests at a probability level of 1 and 5%. Tables and calculations were done using Excel software.

## Figures and Tables

**Table 1:** Mean comparison of sodium selenate (Na_2_SeO_4_) on growth characters of cauliflower cultivars “Clapton” (white) and “Graffiti” (purple). In each column different letters indicate significant differences at the 1% and 5% level based on Duncan’s multiple range tests with three replications.

Cultivar	Treatment (mg/L)	Floret Fresh Weight (g)	Floret Diameter (cm)	Root Fresh Weight (g)	Root Dry Weight (g)	Stem Diameter (cm)	Stem Height (cm)
Clapton	Control	448.49 ± 8.10c	14.41 ± 0.31e	95.66 ± 0.63e	12.33 ± 0.31d	3.18 ± 0.02ed	22.16 ± 1.10c
	5 Na_2_SeO_4_	503.99 ± 8.65ab	15.27 ± 0.16ed	113.83 ± 5.89cde	15.79 ± 0.41cd	3.27 ± 0.02cde	23.58 ± 0.67c
	10 Na_2_SeO_4_	527.45 ± 9.02a	16.24 ± 0.15cd	132.50 ± 2.12c	19.84 ± 0.36c	3.58 ± 0.06b	22.33 ± 0.47c
	15 Na_2_SeO_4_	512.02 ± 27.10ab	15.33 ± 0.27de	119.99 ± 3.14cd	15.49 ± 1.02cd	3.50 ± 0.03bc	21.72 ± 0.31c
	20 Na_2_SeO_4_	469.22 ±5.34bc	15.29 ± 0.33de	108.05 ± 3.54ed	14.49 ± 0.07d	3.35 ± 0.02bcde	21.22 ± 0.17c
Graffiti	Control	241.33 ± 6.76e	15.71 ± 0.01cd	210.22 ± 13.85b	25.55 ± 2.54b	3.12 ± 0.05e	30.97 ± 1.89b
	5 Na_2_SeO_4_	286.89 ± 18.48ed	17.84 ± 0.15a	241.25 ± 3.65a	29.32 ± 2.06b	3.30 ± 0.02bcde	37.10 ± 2.56a
	10 Na_2_SeO_4_	318.11 ± 2.36d	18.33 ± 0.65a	295.78 ± 3.74a	35.65 ± 0.41a	3.92 ± 0.23a	36.00 ± 2.35ab
	15 Na_2_SeO_4_	316.77 ± 7.61d	17.54 ± 0.09ab	248.16 ± 5.11a	30.32 ± 1.36b	3.47 ± 0.02bcd	35.61 ± 1.74ab
	20 Na_2_SeO_4_	273.33 ± 9.03ed	16.47 ± 0.28bc	241.66 ± 2.20a	26.79 ± 2.37b	3.39 ± 0.05bcd	31.71 ± 1.55ab

**Table 2:** Mean comparison of sodium selenate (Na_2_SeO_4_) treatment on growth traits of the Cauliflower cultivars “Clapton” and “Graffiti”. In each column, same letters are not significantly different at the 1% and 5% levels based on Duncan’s multiple range tests with three repetitions.

Cultivar	Treatment (mg/L)	Fresh Weight (g)		Leave Area (cm^2^)	Dry Weight (g)		Root Length (cm)
		Leave	Stem		Leave	Stem	
Clapton	Control	329.99 ± 0.64h	140.99 ± 0.16g	423.82 ± 2.48f	18.33 ± 0.42g	9.16 ± 0.07g	34.58 ± 0.92de
	5 Na_2_SeO_4_	359.41 ± 2.60g	156.24 ± 0.76ef	440.35 ± 0.48e	22.85 ± 0.33f	11.22 ± 0.40f	36.75 ± 1.32cd
	10 Na_2_SeO_4_	405.08 ± 6.42f	160.66 ± 2.56e	473.59 ± 1.03bc	27.22 ± 0.74e	14.08 ± 0.24e	40.66 ± 0.31ab
	15 Na_2_SeO_4_	366.35 ± 4.36g	160.41 ± 1.69e	452.96 ± 1.09ed	23.06 ± 0.12f	10.61 ± 0.20gf	32.5 ± 0.72e
	20 Na_2_SeO_4_	341.94 ± 1.16h	147.91 ± 0.52gf	448.37 ± 1.11e	22.4 ± 0.17f	9.7 ± 0.15gf	32.74 ± 0.67e
Graffiti	Control	575.75 ± 10.52e	197.66 ± 0.79d	455.33 ± 1.33ed	41.4 ± 0.44d	20.33 ± 0.16d	36.88 ± 1.93bcd
	5 Na_2_SeO_4_	607.33 ± 3.52d	213.66 ± 3.20c	466.54 ± 3.62cd	49.78 ± 1.17b	22.78 ± 0.78c	38.27 ± 0.96bcd
	10 Na_2_SeO_4_	728.93 ± 3.05a	288.83 ± 7.44a	488.60 ± 3.55b	55.21 ± 0.45a	28.38 ± 0.54a	42.22 ± 0.80a
	15 Na_2_SeO_4_	708.16 ± 2.80b	261.5 ± 2.16b	535.17 ± 14.01a	46.55 ± 0.67c	25.33 ± 0.44b	40.44 ± 1.84abc
	20 Na_2_SeO_4_	691.01 ± 4.99c	216.16 ± 3.76c	472.13 ± 3.24c	42.63 ± 0.98d	21.74 ± 0.54cd	40.22 ± 0.75abc

**Table 3:** Physiological traits of cauliflower cultivars “Clapton” and “Graffiti” after fortification with sodium selenate (Na_2_SeO_4_). In each column, same letters are not significantly different at the1% and 5% level based on Duncan’s multiple range tests with three repetitions (Chlorophyll and Carotenoid in leaves, Antioxidants, Phenol and Se concentration in floret).

Cultivar	Treatment (mg/L)	Chlorophyll (mg/g FW)			Carotenoid (mg/g FW)	Antioxidants (DPPH %)	Total Polyphenolic (mg Gallic Acid 100 g^−1^)	Se Content (mg/kg DW)
		a	b	Total				
Clapton	Control	0.41 ± 0.00e	0.11 ± 0.00de	0.52 ± 0.01f	0.12 ± 0.00f	14 ± 1.92f	170.6 ± 2.57f	0.03 ± 0.00g
	5 Na_2_SeO_4_	0.54 ± 0.00c	0.14 ± 0.00c	0.68 ± 0.00d	0.15 ± 0.00e	18 ± 0.96ef	178.32 ± 4.62ef	0.12 ± 0.00f
	10 Na_2_SeO_4_	0.70 ± 0.00a	0.16 ± 0.00b	0.87 ± 0.01b	0.21 ± 0.00b	28.5 ± 2.46e	192.48 ± 2.36d	0.23 ± 0.02de
	15 Na_2_SeO_4_	0.74 ± 0.00a	0.20 ± 0.00a	0.95 ± 0.00a	0.25 ± 0.00a	25 ± 0.48ef	202.31 ± 3.30d	0.27 ± 0.03d
	20 Na_2_SeO_4_	0.61 ± 0.00b	0.14 ± 0.00bc	0.76 ± 0.00	0.18 ± 0.00c	16 ± 0.96f	191.63 ± 5.00ed	0.57 ± 0.01a
Graffiti	Control	0.34 ± 0.01f	0.09 ± 0.00e	0.44 ± 0.01g	0.08 ± 0.00g	147 ± 6.72c	249.52 ± 0.66c	0.04 ± 0.00g
	5 Na_2_SeO_4_	0.45 ± 0.01ed	0.10 ± 0.00de	0.55 ± 0.01f	0.14 ± 0.00e	205.66 ± 1.94a	256.02 ± 2.26c	0.18 ± 0.01e
	10 Na_2_SeO_4_	0.60 ± 0.00b	0.14 ± 0.00bc	0.63 ± 0.00e	0.21 ± 0.00b	170.33 ± 0.27b	323.29 ± 4.15a	0.36 ± 0.00c
	15 Na_2_SeO_4_	0.48 ± 0.01d	0.14 ± 0.00bc	0.74 ± 0.00c	0.17 ± 0.00d	145 ± 2.88c	324.66 ± 1.98a	0.40 ± 0.00bc
	20 Na_2_SeO_4_	0.48 ± 0.00d	0.11 ± 0.00d	0.60 ± 0.00e	0.14 ± 0.00e	129 ± 4.82d	305.19 ± 6.23b	0.44 ± 0.00b

## Data Availability

This study did not report any data.
